# Frailty phenotype and long-term mortality after curative colorectal cancer surgery: a retrospective multicenter cohort study

**DOI:** 10.3389/fonc.2026.1913036

**Published:** 2026-10-07

**Authors:** Yuxiao Xia, Minghui Li, Yuanling Zeng, Xiao Hu, Dan Zhou, Yiqun Guo, Meng Zou, Xuewei Yang, Fang Chen, Wei Wang

**Affiliations:** 1 Department of Neurology, Shenzhen Bao’an District Songgang People’s Hospital, Shenzhen, China; 2 School of Innovation and Entrepreneurship Education, Chongqing University of Posts and Telecommunications, Chongqing, China; 3 The Second Affiliated Hospital, Guangzhou Medical University, Guangzhou, China

**Keywords:** colorectal cancer, frailty, long-term mortality, pseudo-observation, restricted mean survival time, retrospective cohort

## Abstract

**Background:**

Frailty is associated with poor short-term surgical outcomes, but whether the Fried frailty phenotype is associated with long-term mortality after curative colorectal cancer (CRC) surgery, independently of age and tumor stage, is uncertain. We examined this association using restricted mean survival time (RMST), which does not require proportional hazards.

**Methods:**

This retrospective multicenter cohort study included 304 patients aged ≥60 years who underwent curative CRC surgery at three centers in China in 2018–2019. The preoperative Fried phenotype was analyzed as robust (n=131), pre-frail (n=57), or frail (n=116); binary (robust versus non-robust) and ordinal codings were secondary. The primary outcome was 5-year all-cause mortality. Because proportional hazards were violated (Schoenfeld test P = 0.011), the RMST difference at 60 months was the primary effect measure, adjusted by pseudo-observation regression for age, sex, and TNM stage, the minimal adjustment set from a directed acyclic graph. Stage-specific and time-varying-effect analyses were also performed.

**Results:**

Over a median follow-up of 60 months, 27 patients (8.9%) died. Five-year survival was 96.2%, 84.2%, and 88.8% in robust, pre-frail, and frail patients (log-rank P = 0.015). Unadjusted RMST differences versus robust patients were −2.38 months (95% CI −4.45 to −0.78) for pre-frail and −2.73 months (−4.58 to −1.09) for frail patients. After adjustment for age, sex, and TNM stage they were −1.48 months (−3.42 to 0.45; P = 0.13) and −0.81 months (−2.56 to 0.93; P = 0.36); for non-robust versus robust patients, −1.16 months (−2.50 to 0.19; P = 0.092). Estimates were in the same direction within every stage but imprecise. The hazard ratio for non-robust patients appeared higher in the first 36 months than later, but period-specific estimates were unstable and did not differ significantly (P = 0.15).

**Conclusions:**

The Fried frailty phenotype identified older patients with lower 5-year survival after curative CRC surgery. After adjustment for age and tumor stage the association was attenuated and imprecise, so an independent effect could not be established with 27 deaths. The apparent early concentration of excess mortality in non-robust patients is hypothesis-generating and requires confirmation in larger cohorts with treatment and cause-of-death data.

## Introduction

Colorectal cancer (CRC) is the second most common malignancy in China, with an estimated 517,100 new cases and 240,000 deaths in 2022 (1). Curative surgical resection is the primary treatment for localized and locally advanced CRC. Although perioperative mortality has declined, long-term survival still varies among patients with similar tumor characteristics (2). Patient-level factors that are associated with long-term mortality beyond tumor stage could inform postoperative surveillance and follow-up.

Frailty is a clinical syndrome of diminished physiological reserves and increased vulnerability to stressors (3). The Fried frailty phenotype, developed in the Cardiovascular Health Study (CHS) (3), defines frailty through five criteria: unintentional weight loss, self-reported exhaustion, weakness, slow walking speed, and low physical activity. Compared with deficit-accumulation indices such as the modified frailty index (mFI), this phenotype-based approach more directly measures physical vulnerability.

Several meta-analyses have shown that frailty is associated with higher short-term morbidity and mortality after CRC surgery (4–6). Davey and Joyce (4) reported a pooled hazard ratio (HR) of 2.95 (95% CI 1.49–5.82) for overall mortality in frail CRC surgical patients, and Moreno-Carmona et al. (5) found an HR of 2.99 (95% CI 2.27–3.93) for overall survival in colon cancer. Most contributing studies used the mFI or administrative frailty measures, focused on 30-day or 90-day outcomes (6), and assumed proportional hazards.

Whether the Fried phenotype is associated with long-term (5-year) mortality after curative CRC surgery, and whether that association persists after accounting for age and tumor stage, is not well studied. The question matters clinically for two reasons. First, frailty is strongly correlated with age and with tumor burden, so an unadjusted association may largely reflect these factors rather than frailty itself. Second, if the association varies over time after surgery, follow-up protocols could in principle be adapted accordingly, although such a time pattern would first need to be established.

The standard Cox proportional hazards model may be inappropriate when the effect varies over time. The restricted mean survival time (RMST), defined as the area under the Kaplan-Meier curve up to a restriction time, is a clinically interpretable alternative that does not require the proportional hazards assumption (7, 8). RMST quantifies the average survival time difference between groups, which can be communicated directly to patients.

We conducted a retrospective multicenter cohort study to evaluate the association between the Fried frailty phenotype and 5-year all-cause mortality after curative CRC surgery, using RMST as the primary effect measure and pseudo-observation regression for covariate adjustment.

## Materials and methods

### Study design and participants

This was a retrospective multicenter cohort study conducted at three university-affiliated hospitals in China. Patients who underwent surgery between January 2018 and December 2019 were identified from institutional surgical and geriatric assessment records. Inclusion criteria were: (1) age ≥60 years; (2) histologically confirmed colorectal adenocarcinoma; (3) elective curative surgical resection, defined as an R0 resection with no macroscopic or microscopic residual tumor; and (4) a completed preoperative Fried frailty assessment, which was part of the routine preoperative geriatric evaluation at the participating centers. Exclusion criteria were: (1) emergency surgery without a standard preoperative assessment; (2) metastatic disease (stage IV) at diagnosis; (3) a history of any other malignancy; and (4) inability to provide informed consent. Patients who could not complete the physical performance components of the frailty assessment (grip strength or walking test) because of acute illness, immobility, or severe cognitive impairment were not eligible, because a frailty score could not be assigned.

Two consequences of these criteria are important for interpretation. First, patients who were too unwell to complete a performance-based frailty assessment are likely to be among the most frail and at the highest risk of death; their exclusion would therefore tend to bias the frailty-mortality association toward the null and to underestimate the true prognostic gradient. Second, because all participants were selected for curative surgery, the cohort represents older patients judged fit enough for resection, so the results do not apply to patients managed non-operatively.

The study was approved by the institutional review boards of all participating centers and conducted in accordance with the Declaration of Helsinki. All participants provided written informed consent for the use of their clinical data.

### Frailty assessment

The Fried frailty phenotype (3) was assessed within 7 days before surgery by research nurses trained in a standardized protocol. The five criteria were operationalized as in the original CHS definition (**Supplementary Table 1**): (1) unintentional weight loss, defined as self-reported loss of more than 4.5 kg or more than 5% of body weight in the preceding year without deliberate dieting; (2) self-reported exhaustion, defined as answering “a moderate amount of the time (3–4 days)” or “most of the time” to either of two items from the Center for Epidemiologic Studies Depression scale (“I felt that everything I did was an effort” and “I could not get going”); (3) weakness, defined as dominant-hand grip strength below the CHS sex- and body mass index (BMI)-specific cutoffs (men: ≤29 kg for BMI ≤24, ≤30 kg for BMI 24.1–28, ≤32 kg for BMI >28; women: ≤17 kg for BMI ≤23, ≤17.3 kg for BMI 23.1–26, ≤18 kg for BMI 26.1–29, ≤21 kg for BMI >29), measured with a handheld electronic dynamometer with the patient seated and the elbow flexed at 90°; (4) slowness, defined as time to walk 4.57 m (15 feet) at usual pace above the CHS sex- and height-specific cutoffs (men: ≥7 s for height ≤173 cm, ≥6 s for height >173 cm; women: ≥7 s for height ≤159 cm, ≥6 s for height >159 cm); and (5) low physical activity, defined as weekly energy expenditure below the CHS sex-specific cutoff (<383 kcal/week for men and <270 kcal/week for women) estimated from a structured physical activity questionnaire. The original CHS cutoffs were applied without recalibration to Chinese normative data; population-specific thresholds [for example, the Asian Working Group for Sarcopenia grip strength cutoffs of <28 kg for men and <18 kg for women (9)] would classify some patients differently, and we address this in the Limitations.

Each criterion was scored 0 or 1 and summed. Following Fried et al. (3), patients were classified as robust (0 criteria), pre-frail (1–2 criteria), or frail (≥3 criteria). This three-category classification was the primary exposure. Two secondary codings were also analyzed: a binary classification (robust versus non-robust, the latter combining pre-frail and frail patients), which had been used as the primary exposure in the previously submitted version of this analysis, and an ordinal coding (0, 1, 2 for robust, pre-frail, and frail) to test for a monotonic trend. The binary classification was not prespecified in a protocol and was originally motivated by the similar survival of pre-frail and frail patients; it is therefore presented as an exploratory analysis. The individual criterion count (0–5) was not retained in the analytic dataset, so the score could not be analyzed as a continuous variable.

### Outcome, follow-up, and censoring

The primary outcome was all-cause mortality within 60 months of surgery. Follow-up visits were scheduled every 3 months during the first 2 postoperative years and every 6 months thereafter, in line with CRC surveillance guidelines (10), and were conducted through outpatient visits or telephone calls with the patient or a family member. Vital status was ascertained from hospital records, follow-up contacts, and linkage to regional vital statistics registries. Survival time was calculated from the date of surgery to the date of death, the date of last contact for patients lost to follow-up, or 60 months after surgery, whichever came first; survivors were administratively censored at 60 months. Of the 304 patients, 303 (99.7%) were followed to death or to 60 months; one patient was last known to be alive at 50 months and was censored at that time. The median follow-up by the reverse Kaplan-Meier method was 60 months. Cause of death, recurrence, postoperative complications, and adjuvant treatment were not available in the analytic dataset, so this analysis is restricted to all-cause mortality.

### Covariates and causal framework

Covariates were selected using a directed acyclic graph (DAG) constructed before the revised analysis (**Supplementary Figure 1**) (11). Age, sex, comorbidity (diabetes mellitus, coronary heart disease, and hypertension), and TNM stage were considered confounders, because each can cause both frailty (for example, tumor burden causes weight loss and exhaustion) and mortality. TNM stage also determines selection for curative surgery jointly with frailty. Because cohort entry was conditional on surgery, the path from frailty through surgical selection to TNM stage and mortality is already open by design; adjusting for TNM stage closes this path rather than opening a new one, and the minimal sufficient adjustment set identified with the dagitty package was {age, sex, comorbidity, TNM stage} (11, 12). Hemoglobin and albumin were treated as potential mediators (consequences of both frailty and tumor burden) and adjuvant therapy as an unmeasured mediator. Geriatric syndromes that overlap with the frailty construct (malnutrition, functional dependence) or are plausibly downstream of it (anxiety, depression, chronic pain, poor sleep) were not included as covariates, to avoid overadjustment (13). TNM stage was classified according to the 8th edition of the AJCC staging system.

### Statistical analysis

#### Descriptive statistics

Continuous variables are presented as mean ± standard deviation or median (interquartile range) and were compared across the three frailty categories with one-way analysis of variance or the Kruskal-Wallis test. Categorical variables are presented as frequencies (percentages) and were compared with the chi-squared test or Fisher’s exact test.

#### Kaplan-Meier analysis

Survival curves were estimated with the Kaplan-Meier method and compared with the log-rank test (overall, pairwise, and a log-rank test for trend across the ordinal categories). Survival probabilities at 12, 24, 36, 48, and 60 months are reported with 95% confidence intervals as descriptive milestones; no time point was designated as a landmark.

#### Proportional hazards assessment

The proportional hazards assumption was tested with scaled Schoenfeld residuals (14) using Kaplan-Meier-transformed, log-transformed, and untransformed time.

#### Restricted mean survival time

RMST was estimated as the area under the Kaplan-Meier curve up to the restriction time τ (7). The primary restriction time was τ=60 months, the end of follow-up; RMST differences at τ=24, 36, and 48 months are reported to describe how the difference accumulated over time. Differences are consistently expressed as the comparison group minus the robust group, so that negative values indicate shorter survival in the comparison group. Confidence intervals for unadjusted differences were obtained from 2000 stratified bootstrap replicates, and Greenwood-based standard errors were used as a check.

#### Pseudo-observation regression

Covariate-adjusted RMST differences were estimated with the pseudo-observation approach (15, 16). For each patient, a jackknife pseudo-observation of the RMST at τ was computed, and the pseudo-observations were regressed on the exposure and covariates using a linear model with Huber-White (HC1) robust standard errors. The primary model included the three-category frailty variable, age, sex, and TNM stage (model M2). Nested models were fitted to show how the estimate changed with adjustment: unadjusted (M0); age and sex (M1, the primary model of the previously submitted version); M2 plus diabetes, coronary heart disease, and hypertension (M3, the full DAG-based adjustment set); M3 plus hemoglobin and albumin (M4, which conditions on potential mediators and is shown for completeness only); and M3 plus log-transformed carcinoembryonic antigen (M5). A 2-degree-of-freedom Wald test was used for the joint effect of frailty in the primary model. The same models were fitted for the binary and ordinal codings. Because pseudo-observation regression is a linear model, the events-per-variable rule for Cox regression does not apply directly, but with 27 deaths all adjusted estimates are imprecise and are interpreted as such.

#### Stage-specific and stage-stratified analyses

To address whether the association differed by tumor stage and to avoid relying solely on additive adjustment, we repeated the binary comparison within each TNM stage (unadjusted and adjusted for age and sex, with pseudo-observations computed within stage), in stages I and II combined, and in a model with stage-stratified pseudo-observations. Log-rank tests stratified by stage were also performed. Stage III contained only three robust patients, so stage-specific estimates in this stratum are reported for transparency but are not interpretable.

#### Time-varying effect analyses

To examine whether the association varied over follow-up without assuming a change point, we (a) plotted the smoothed scaled Schoenfeld residuals as a time-varying log HR; (b) fitted a Cox model with a frailty × log(time) interaction; and (c) fitted an Aalen additive hazards model (17). To describe the pattern in a form comparable with the previously submitted version, period-specific HRs for 0–36 and 36–60 months were estimated with a piecewise exponential model. Because no robust patient died before 36 months, standard maximum likelihood estimates for the early period are infinite; we therefore used Firth-type mean bias reduction (18, 19), and we tested whether the two period-specific HRs differed. The 36-month cut point corresponds to the conventional 3-year survival milestone in CRC trials, but we emphasize that it was not prespecified as a change point and that the analysis is descriptive.

#### Sensitivity to unmeasured confounding

Because adjuvant therapy was not recorded, we computed E-values (20) for the risk ratio of 5-year death (non-robust versus robust) from a modified Poisson model adjusted for age, sex, and TNM stage. Conventional and Firth-penalized Cox HRs are reported in the supplement for comparison with other studies, with the caveat that they average over a period of non-proportional hazards.

#### Missing data

All analysis variables (frailty category, vital status, survival time, age, sex, TNM stage, comorbidities, hemoglobin, albumin, and CEA) were complete for all 304 included patients, so no imputation was required.

All analyses were performed in R version 4.3.1 (R Foundation for Statistical Computing, Vienna, Austria) with the survival, sandwich, brglm2, coxphf, and dagitty packages. Two-sided P values are reported without adjustment for multiplicity; given the number of deaths, all results are interpreted as estimates with confidence intervals rather than as hypothesis tests. This study is reported in accordance with the STROBE guidelines (21).

## Results

### Study population and follow-up

A total of 304 patients were included [mean age 68.6 ± 5.3 years; 157 (51.6%) male]. By the Fried phenotype, 131 (43.1%) were robust, 57 (18.8%) pre-frail, and 116 (38.2%) frail. Frailty category was associated with older age, lower BMI, lower hemoglobin and albumin, higher CEA, greater comorbidity burden, more geriatric syndromes, and more advanced TNM stage (**Table 1**). Stage I disease was present in 86.3% of robust, 66.7% of pre-frail, and 40.5% of frail patients, and stage III in 2.3%, 10.5%, and 19.0%, respectively (P<0.001).

**Table 1:** Baseline characteristics by Fried frailty category.

Characteristic	Robust (n=131)	Pre-frail (n=57)	Frail (n=116)	P value
Age, years, mean ± SD	65.1 ± 3.7	68.8 ± 5.1	72.5 ± 4.0	<0.001
BMI, kg/m², mean ± SD	23.1 ± 2.0	22.7 ± 2.2	21.2 ± 2.4	<0.001
Hemoglobin, g/L, mean ± SD	122.2 ± 11.3	117.7 ± 14.2	105.9 ± 16.3	<0.001
Albumin, g/L, mean ± SD	38.3 ± 5.2	36.9 ± 3.9	33.9 ± 4.3	<0.001
CEA, ng/mL, median (IQR)	38.1 (34.9–43.8)	42.8 (36.8–67.8)	65.1 (37.9–78.4)	<0.001
Charlson comorbidity index, median (IQR)	0 (0–1)	1 (1–2)	2 (1–2)	<0.001
Male sex, n (%)	66 (50.4)	34 (59.6)	57 (49.1)	0.399
Hypertension, n (%)	32 (24.4)	6 (10.5)	30 (25.9)	0.057
Diabetes mellitus, n (%)	15 (11.5)	12 (21.1)	31 (26.7)	0.009
Coronary heart disease, n (%)	16 (12.2)	12 (21.1)	30 (25.9)	0.022
Malnutrition, n (%)	12 (9.2)	13 (22.8)	64 (55.2)	<0.001
Anxiety, n (%)	3 (2.3)	7 (12.3)	29 (25.0)	<0.001
Depression, n (%)	2 (1.5)	7 (12.3)	27 (23.3)	<0.001
Chronic pain, n (%)	13 (9.9)	24 (42.1)	68 (58.6)	<0.001
Poor sleep, n (%)	14 (10.7)	26 (45.6)	94 (81.0)	<0.001
Cognitive impairment, n (%)	2 (1.5)	9 (15.8)	49 (42.2)	<0.001
Functional dependence, n (%)	17 (13.0)	30 (52.6)	81 (69.8)	<0.001
TNM stage, n (%)				<0.001
Stage I	113 (86.3)	38 (66.7)	47 (40.5)	
Stage II	15 (11.5)	13 (22.8)	47 (40.5)	
Stage III	3 (2.3)	6 (10.5)	22 (19.0)	
Death within 5 years, n (%)	5 (3.8)	9 (15.8)	13 (11.2)	0.012

P values from one-way analysis of variance, Kruskal-Wallis test, chi-squared test, or Fisher’s exact test as appropriate. BMI, body mass index; CEA, carcinoembryonic antigen.

During 17,685 person-months of follow-up, 27 patients (8.9%) died: 5 (3.8%) robust, 9 (15.8%) pre-frail, and 13 (11.2%) frail patients (P = 0.012). Follow-up to 60 months or death was complete for 303 patients (99.7%); one robust patient with stage I disease was censored alive at 50 months.

### Unadjusted survival

Five-year survival was 96.2% (95% CI 92.9–99.5) in robust, 84.2% (75.3–94.2) in pre-frail, and 88.8% (83.2–94.7) in frail patients (overall log-rank P = 0.015; P for trend 0.032; **Figure 1A**; **Supplementary Table 2**). Both non-robust categories had lower survival than robust patients (pre-frail versus robust P = 0.003; frail versus robust P = 0.023), whereas pre-frail and frail patients did not differ from each other (P = 0.44). Combining the two categories, 5-year survival was 87.3% (82.5–92.4) in non-robust patients (log-rank P = 0.006 versus robust; **Supplementary Figure 2**). Survival also differed by TNM stage (**Supplementary Figure 3**). When the log-rank test was stratified by TNM stage, the frailty association was attenuated (P = 0.071 for three categories; P = 0.086 for the binary comparison).

**Figure 1:**
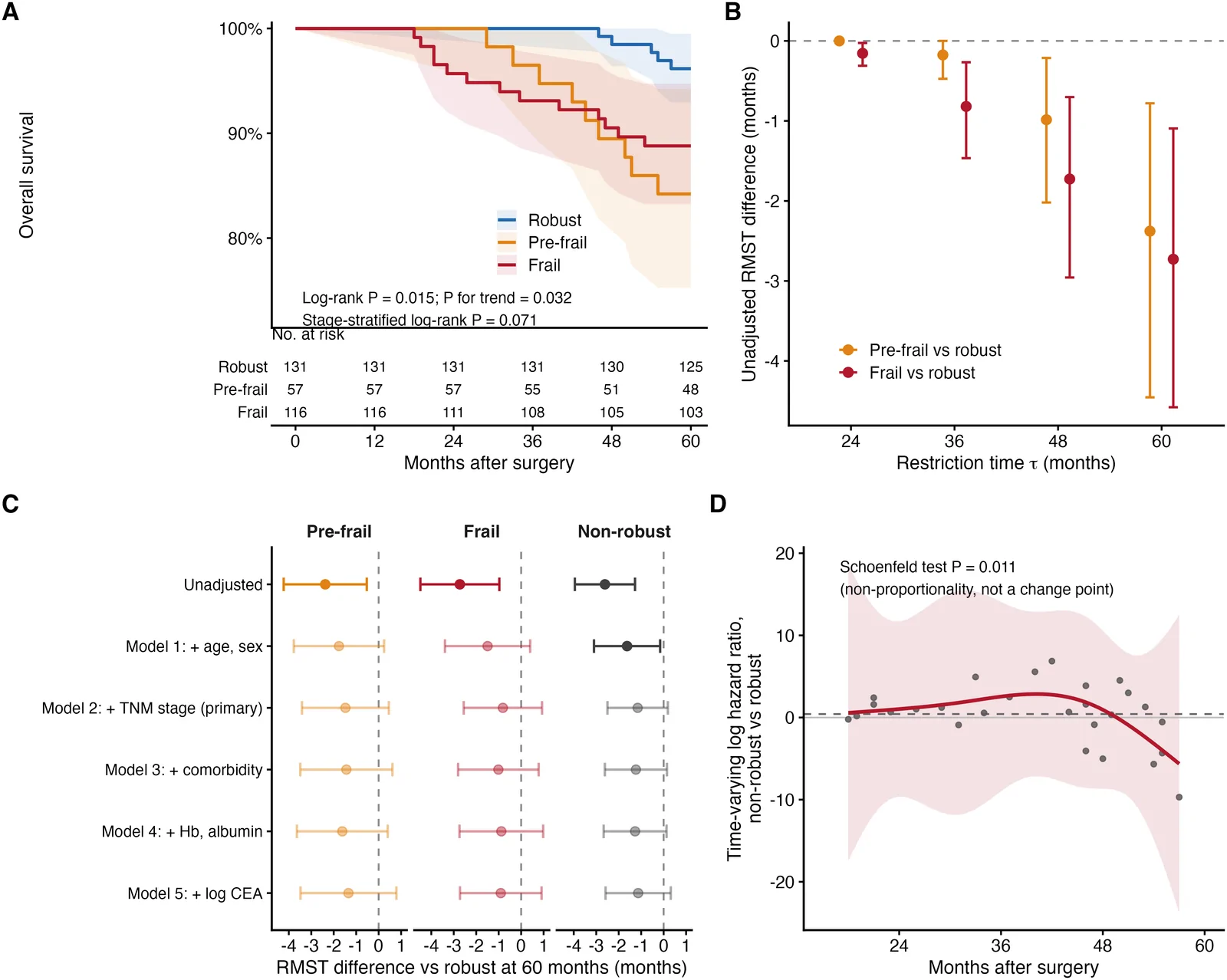
Frailty phenotype and survival after curative colorectal cancer surgery. **(A)** Kaplan-Meier overall survival curves for robust (blue, n=131), pre-frail (orange, n=57), and frail (red, n=116) patients, with 95% confidence bands and the number at risk; overall log-rank P = 0.015, log-rank test for trend P = 0.032. **(B)** Unadjusted restricted mean survival time (RMST) differences versus robust patients at restriction times τ=24, 36, 48, and 60 months. Points are the RMST difference (comparison group minus robust; negative values indicate shorter survival in the comparison group) and error bars are 95% confidence intervals from 2000 stratified bootstrap replicates. **(C)** Covariate-adjusted RMST differences at τ=60 months from pseudo-observation regression with robust standard errors, for pre-frail versus robust, frail versus robust, and non-robust (pre-frail plus frail) versus robust patients. Models are nested: unadjusted; age and sex; age, sex, and TNM stage (primary model); plus diabetes, coronary heart disease, and hypertension (M3); M3 plus hemoglobin and albumin; and M3 plus log CEA. Error bars are 95% confidence intervals. **(D)** Time-varying log hazard ratio for non-robust versus robust patients estimated from smoothed scaled Schoenfeld residuals of a Cox model adjusted for age, sex, and TNM stage (untransformed time). Points are scaled Schoenfeld residuals at each death time, the solid line is a natural spline smooth (4 degrees of freedom), the shaded band is ±2 standard errors and extends beyond the plotted range, the dashed line is the time-averaged Cox log hazard ratio, and the grey line marks zero. Scaled Schoenfeld residual test P = 0.011.

The RMST over 60 months was 59.69 months (SE 0.15) in robust, 57.32 months (0.93) in pre-frail, and 56.97 months (0.88) in frail patients. Unadjusted RMST differences versus robust patients were −2.38 months (95% CI −4.45 to −0.78; P = 0.012) for pre-frail and −2.73 months (−4.58 to −1.09; P = 0.003) for frail patients, and −2.61 months (−4.02 to −1.38; P<0.001) for the combined non-robust group (**Table 2**). Each step up the ordinal scale was associated with −1.38 months (−2.24 to −0.51). The differences accumulated with longer restriction time: at τ=36 months they were −0.18 months (−0.47 to 0.00) for pre-frail and −0.82 months (−1.47 to −0.27) for frail patients (**Figure 1B**; **Supplementary Table 3**).

**Table 2:** Unadjusted 5-year survival and restricted mean survival time by frailty category.

Group	n/deaths	5-year survival, % (95% CI)	RMST at 60 months, months (SE)	RMST difference vs robust, months (95% CI)	P value
Robust	131/5	96.2 (92.9–99.5)	59.69 (0.15)	Reference	
Pre-frail	57/9	84.2 (75.3–94.2)	57.32 (0.93)	-2.38 (-4.45 to -0.78)	0.012
Frail	116/13	88.8 (83.2–94.7)	56.97 (0.88)	-2.73 (-4.58 to -1.09)	0.003
Non-robust (pre-frail + frail)	173/22	87.3 (82.5–92.4)	57.08	-2.61 (-4.02 to -1.38)	<0.001

RMST, restricted mean survival time (area under the Kaplan-Meier curve to 60 months); SE, Greenwood-based standard error. Differences are comparison group minus robust; confidence intervals from 2000 stratified bootstrap replicates.

### Covariate-adjusted RMST differences

Adjustment progressively attenuated the estimates (**Table 3**; **Figure 1C**). After adjustment for age and sex, the RMST differences were −1.77 months (95% CI −3.78 to 0.24; P = 0.085) for pre-frail and −1.50 months (−3.39 to 0.40; P = 0.12) for frail patients; for the binary coding the difference was −1.63 months (−3.10 to −0.16; P = 0.030). In the primary model, which additionally adjusted for TNM stage, the differences were −1.48 months (−3.42 to 0.45; P = 0.13) for pre-frail and −0.81 months (−2.56 to 0.93; P = 0.36) for frail patients (joint 2-df P = 0.23), −0.43 months per ordinal category (−1.29 to 0.44; P = 0.33), and −1.16 months (−2.50 to 0.19; P = 0.092) for non-robust versus robust patients. Full coefficients of the primary model are given in **Supplementary Table 4**. Further adjustment for diabetes, coronary heart disease, and hypertension changed the estimates little (non-robust versus robust −1.23 months, −2.62 to 0.15), as did the addition of hemoglobin and albumin (−1.27 months, −2.67 to 0.13) or log CEA (−1.13 months, −2.59 to 0.32). Adjusted differences at τ=36 and 48 months followed the same pattern (**Supplementary Table 5**).

**Table 3:** Covariate-adjusted RMST differences at 60 months (pseudo-observation regression).

Model	Pre-frail vs robust	Frail vs robust	Per category increase (ordinal)	Non-robust vs robust
Unadjusted	-2.38 (-4.23 to -0.53); P = 0.012	-2.73 (-4.49 to -0.97); P = 0.002	-1.38 (-2.24 to -0.51); P = 0.002	-2.61 (-3.95 to -1.27); P=<0.001
Age, sex	-1.77 (-3.78 to 0.24); P = 0.085	-1.50 (-3.39 to 0.40); P = 0.122	-0.76 (-1.71 to 0.18); P = 0.114	-1.63 (-3.10 to -0.16); P = 0.030
Age, sex, TNM stage (primary)	-1.48 (-3.42 to 0.45); P = 0.134	-0.81 (-2.56 to 0.93); P = 0.361	-0.43 (-1.29 to 0.44); P = 0.332	-1.16 (-2.50 to 0.19); P = 0.092
Age, sex, TNM stage, diabetes, CHD, hypertension	-1.44 (-3.49 to 0.61); P = 0.169	-1.01 (-2.81 to 0.78); P = 0.268	-0.52 (-1.41 to 0.38); P = 0.259	-1.23 (-2.62 to 0.15); P = 0.080
M3 + hemoglobin, albumin (potential mediators)	-1.62 (-3.64 to 0.40); P = 0.116	-0.88 (-2.74 to 0.98); P = 0.353	-0.46 (-1.38 to 0.46); P = 0.326	-1.27 (-2.67 to 0.13); P = 0.075
M3 + log(CEA)	-1.34 (-3.48 to 0.79); P = 0.217	-0.91 (-2.72 to 0.91); P = 0.328	-0.46 (-1.37 to 0.45); P = 0.321	-1.13 (-2.59 to 0.32); P = 0.126

Values are RMST differences at τ = 60 months (comparison group minus robust) with 95% confidence intervals from pseudo-observation regression with HC1 robust standard errors. M3, adjusted for age, sex, TNM stage, diabetes, coronary heart disease, and hypertension. CHD, coronary heart disease; CEA, carcinoembryonic antigen.

### Stage-specific and stage-stratified analyses

Within each TNM stage, non-robust patients had a shorter unadjusted RMST than robust patients: −1.11 months (95% CI −2.39 to −0.09) in stage I, −3.53 months (−6.53 to −0.83) in stage II, and −4.96 months (−9.36 to −1.43) in stage III (**Table 4**; **Supplementary Figure 4**). After adjustment for age and sex within stage, the point estimates remained negative but the confidence intervals included zero in every stratum (stage I −0.83 months, −2.25 to 0.60; stage II −2.28 months, −5.08 to 0.52; stage III −4.62 months, −11.22 to 1.97, based on three robust patients). In stages I and II combined (273 patients, 21 deaths), the unadjusted difference was −2.21 months (−3.65 to −0.95) and the age-, sex-, and stage-adjusted difference −1.01 months (−2.25 to 0.22; P = 0.11). The model with stage-stratified pseudo-observations gave −1.16 months (−2.50 to 0.19), identical to the additively adjusted primary estimate.

**Table 4:** Stage-specific and stage-stratified analyses (non-robust vs robust, RMST at 60 months).

Stratum	Robust, n (deaths)	Non-robust, n (deaths)	Log-rank P	Unadjusted RMST difference, months (95% CI)	Age- and sex-adjusted RMST difference, months (95% CI); P
Stage I	113 (3)	85 (7)	0.073	-1.11 (-2.39 to -0.09)	-0.83 (-2.25 to 0.60); P = 0.254
Stage II	15 (2)	60 (9)	0.800	-3.53 (-6.53 to -0.83)	-2.28 (-5.08 to 0.52); P = 0.111
Stage III	3 (0)	28 (6)	0.400	-4.96 (-9.36 to -1.43)	-4.62 (-11.22 to 1.97); P = 0.170
Stage I + II	128 (5)	145 (16)	0.025	-2.21 (-3.65 to -0.95)	-1.01 (-2.25 to 0.22); P = 0.107
All stages, stratified by TNM	131 (5)	173 (22)	0.086 (stratified)	—	-1.16 (-2.50 to 0.19); P = 0.092

Within-stage pseudo-observations were computed separately in each stratum. The stage III stratum contains three robust patients and no deaths among them; its estimates are shown for transparency only. The stratified-pseudo-observation model uses pseudo-observations computed within TNM stage and additionally adjusts for age and sex.

### Time-varying association

The scaled Schoenfeld residual test indicated non-proportional hazards for frailty (P = 0.011 with Kaplan-Meier-transformed time, 0.029 with log time, and 0.016 with untransformed time; **Supplementary Table 6**). The smoothed time-varying log HR was above zero during the first 3 years and declined thereafter (**Figure 1D**), the frailty × log(time) interaction was negative (P = 0.083), and the Aalen additive model showed the excess hazard accumulating mainly between 18 and 48 months (**Supplementary Figure 5**). Descriptively, no robust patient died before 46 months (median time to death among the 5 robust decedents 54 months), whereas 10 of 22 non-robust deaths occurred within 36 months (median 44 months among pre-frail and 31 months among frail decedents); we note that comparisons restricted to decedents condition on the outcome and are not used for inference.

In the piecewise model with Firth-type bias reduction, the unadjusted HR for non-robust versus robust patients was 16.2 (95% CI 0.95 to 276) in months 0–36 (0 versus 10 deaths) and 1.87 (0.69 to 5.11) in months 36–60 (5 versus 12 deaths); after adjustment for age, sex, and TNM stage the corresponding HRs were 7.0 (0.42 to 116) and 0.82 (0.27 to 2.53). The ratio of the two period-specific HRs was 8.5 (0.46 to 157; P = 0.15). The data are therefore compatible with a larger early association, but the period-specific estimates are unstable and a specific change point cannot be established (**Supplementary Figure 6**).

### Hazard ratios and sensitivity to unmeasured confounding

For comparison with other studies, the conventional Cox HR for non-robust versus robust patients was 3.56 (95% CI 1.35 to 9.39) unadjusted and 1.53 (0.49 to 4.79) after adjustment for age, sex, and TNM stage (Firth-penalized 1.44, 0.50 to 4.79); these average over a period in which the hazards were not proportional (**Supplementary Table 6**). The unadjusted risk ratio for 5-year death was 3.33 (E-value 6.12); the adjusted risk ratio was 1.49 (0.41 to 5.41; E-value 2.34, with an E-value of 1.0 for the confidence limit; **Supplementary Table 7**). An unmeasured confounder such as adjuvant therapy would need to be associated with both frailty and death by a risk ratio of about 2.3 to explain away the adjusted point estimate, and a much weaker one would suffice to move the confidence interval across the null.

## Discussion

In this cohort of 304 older patients who underwent curative CRC surgery, the Fried frailty phenotype identified patients with lower 5-year survival: pre-frail and frail patients lost on average 2.4 and 2.7 months of survival over 5 years compared with robust patients. Much of this difference was accounted for by the older age and more advanced tumor stage of non-robust patients. After adjustment for age, sex, and TNM stage, the point estimates still favored shorter survival in non-robust patients, in every stage stratum and under every adjustment set, but the confidence intervals included zero. With 27 deaths, the study can neither confirm nor exclude an independent association of the magnitude reported in meta-analyses of short-term outcomes (4, 5); a confounder-adjusted RMST difference of anywhere between about 2.5 months of survival lost and 0.2 months gained is compatible with our data. We therefore regard the findings as exploratory.

The revision of this analysis illustrates how the choice of exposure coding and covariates affects conclusions in a small cohort. In the previously submitted version, the exposure was dichotomized as robust versus non-robust, and the model adjusted for age and sex but not TNM stage, on the argument that stage might act as a collider in a surgery-selected cohort. Both choices favored a stronger association. The dichotomy was not prespecified, and merging categories on the basis of an observed lack of difference is a form of data-driven categorization that can exaggerate effects and obscure dose-response (22). We now present the conventional three categories as the primary exposure and the binary and ordinal codings as secondary analyses; the three-category analysis shows no monotonic gradient, with pre-frail patients faring at least as poorly as frail patients, which may reflect chance in small groups, classification error, or the exclusion of the most frail patients before cohort entry. With respect to TNM stage, the DAG makes clear that the collider in a surgical cohort is surgical selection itself, on which the cohort is already conditioned; stage is a cause of both frailty and death, and adjustment for it is required for confounding control. The attenuation of the estimate after stage adjustment is most parsimoniously interpreted as removal of confounding by tumor burden, although we cannot distinguish confounding from mediation (tumor burden causing weight loss that is scored as frailty) or from residual selection effects, and we no longer characterize the attenuation as evidence of collider bias.

The time pattern of the association deserves cautious comment. The proportional hazards assumption was violated, the smoothed time-varying log HR was highest in the first 3 years, and no robust patient died before 46 months. The 18–36 month period in which non-robust deaths clustered coincides with the period of highest recurrence hazard after curative resection (23, 24), and it is biologically plausible that patients with diminished physiological reserve (25, 26) are less able to survive recurrence or its treatment. However, the period-specific HRs were highly unstable, their ratio was not statistically significant, the 36-month cut point was not prespecified, and we lack recurrence and cause-of-death data with which to test the mechanism. We therefore present the early concentration of excess mortality as a hypothesis for larger cohorts rather than as a “vulnerability window” established by these data. Similarly, the comparison of time to death among patients who died, which we had previously reported, conditions on a post-baseline outcome and is now reported only descriptively.

Treatment-related confounding and mediation require particular attention. Adjuvant chemotherapy improves survival in stage III and selected stage II disease, and older and frail patients are less likely to receive it and to complete it (27–29). In our DAG, adjuvant therapy lies on a pathway from frailty to mortality, so a total-effect estimate appropriately does not adjust for it; but if frail patients received less adjuvant therapy, part of the observed association would represent under-treatment rather than frailty itself, which has different implications for intervention. Adjuvant therapy could also act as a confounder if unmeasured factors such as center practice, socioeconomic status, or patient preference influenced both frailty classification and treatment. The E-value analysis indicates that a confounder associated with both frailty and death by a risk ratio of about 2.3 could explain the adjusted point estimate, and that the confidence interval already includes the null; treatment data are therefore essential for any future study of this question.

Our findings should be compared with the literature with these caveats in mind. The unadjusted Cox HR of 3.56 for non-robust versus robust patients is similar to the pooled HRs of 2.95 and 2.99 reported in meta-analyses (4, 5), most of whose contributing studies used the mFI, focused on short-term outcomes (30), and varied in their adjustment for stage. Our adjusted estimates are smaller and imprecise. Whether the Fried phenotype, which captures physical vulnerability, carries prognostic information for long-term mortality beyond age and stage in surgically selected patients remains open. Methodologically, we used RMST because the proportional hazards assumption was violated (7, 8, 31) and pseudo-observation regression (15, 16) to adjust the RMST difference for covariates; these methods yield estimates that are interpretable as average months of survival lost and that do not depend on proportional hazards.

The clinical implications of this study are limited to prognostic description. Preoperative frailty assessment identified a group of older patients with lower 5-year survival, and this information may be useful when discussing prognosis and planning follow-up. Our data do not show that intensified surveillance or frailty-targeted interventions during any postoperative period would improve survival; that question requires interventional studies. Current ASCO guidance recommends geriatric assessment to guide systemic therapy decisions (32), and geriatric-assessment-guided management reduced treatment toxicity in the GAP70+ trial (33). Prehabilitation reduces some short-term complications in frail cancer patients (34, 35), although the trial by Carli et al. (36) found no benefit on 30-day complications in frail CRC patients within an enhanced recovery pathway. Whether interventions that sustain function and nutrition after surgery affect long-term survival in non-robust patients is untested.

This study has strengths: a multicenter cohort assessed with the performance-based Fried phenotype rather than an administrative proxy, near-complete 5-year follow-up, a DAG-based adjustment strategy, and effect measures that do not assume proportional hazards. The limitations are substantial. First, with 27 deaths and 5 deaths among robust patients, all adjusted estimates are imprecise, period-specific estimates are unstable, and the study is best regarded as hypothesis-generating. Second, patients unable to complete the performance-based frailty assessment were not eligible, and all patients were selected for surgery; both exclusions remove the most vulnerable patients and are expected to bias the association toward the null and to limit generalizability. Third, the CHS cutoffs for grip strength, walking speed, and physical activity were applied without recalibration to Chinese normative data (9, 37); misclassification of frailty status is possible and, if non-differential, would also bias toward the null, but the atypically high proportion of frail patients (38.2%) may partly reflect this. Fourth, the individual criterion count was not retained, so a continuous score could not be analyzed. Fifth, cause of death, recurrence, postoperative complications, and adjuvant treatment were not available, which prevents mechanistic interpretation and leaves treatment-related confounding and mediation unaddressed. Sixth, the analytic strategy (RMST, the three-category primary exposure, and the covariate set) was not prespecified in a registered protocol; the current analysis plan was fixed in response to peer review before the revised analyses were run, and all prespecified and exploratory analyses are reported. Seventh, the cohort consisted of Chinese patients from three centers, and generalizability to other populations and health systems is unknown. Finally, the observational design cannot establish causation, and unmeasured factors such as socioeconomic status and caregiver support may contribute to the association.

## Conclusion

In this retrospective multicenter cohort of older patients undergoing curative CRC surgery, the Fried frailty phenotype identified patients with lower 5-year survival, but after adjustment for age, sex, and TNM stage the association was attenuated and imprecise, and an independent prognostic effect could not be established. The excess mortality among non-robust patients appeared to be concentrated in the first 3 postoperative years, a pattern that is hypothesis-generating. Larger cohorts with recorded treatment, recurrence, and cause of death, and with population-specific frailty cutoffs, are needed to determine whether the frailty phenotype adds prognostic information beyond age and stage and whether any postoperative period warrants frailty-specific care.

## Data Availability

The raw data supporting the conclusions of this article will be made available by the authors, without undue reservation.
