# Supplementary material for: Frailty phenotype and long-term mortality after curative colorectal cancer surgery: a retrospective multicenter cohort study
Source: Front Oncol. 2026 Oct 7;16:1913036. doi: 10.3389/fonc.2026.1913036 (PMC13682989; doi:10.3389/fonc.2026.1913036)
Supplement: Supplementary file 1 [file DataSheet1.pdf]

Supplementary Material

Frailty Phenotype and Long-Term Mortality After Curative Colorectal Cancer Surgery: A Retrospective Multicenter Cohort Study

Supplementary Tables

Supplementary Table 1. Operationalization of the Fried frailty phenotype criteria (Cardiovascular Health Study cutoffs, applied without recalibration)

Criterion	Measurement in this study	Cutoff defining the criterion as present
Shrinking	Self-reported unintentional weight loss over the preceding year; current weight measured at assessment	Loss of >4.5 kg or >5% of body weight, not due to deliberate dieting
Exhaustion	Two CES-D items (“I felt that everything I did was an effort”; “I could not get going”), frequency in the past week	Either item answered “a moderate amount of the time (3–4 days)” or “most of the time”
Weakness	Dominant-hand grip strength, handheld electronic dynamometer, seated with elbow flexed at 90°	Men: ≤29 kg (BMI ≤24), ≤30 kg (BMI 24.1–28), ≤32 kg (BMI >28). Women: ≤17 kg (BMI ≤23), ≤17.3 kg (BMI 23.1–26), ≤18 kg (BMI 26.1–29), ≤21 kg (BMI >29)
Slowness	Time to walk 4.57 m (15 feet) at usual pace	Men: ≥7 s (height ≤173 cm), ≥6 s (height >173 cm). Women: ≥7 s (height ≤159 cm), ≥6 s (height >159 cm)
Low physical activity	Weekly energy expenditure estimated from a structured physical activity questionnaire	Men: <383 kcal/week. Women: <270 kcal/week

Classification: 0 criteria, robust; 1–2 criteria, pre-frail; ≥3 criteria, frail. CES-D, Center for Epidemiologic Studies Depression scale; BMI, body mass index.

Supplementary Table 2. Kaplan-Meier survival probabilities at 12 to 60 months by frailty category

Group	Months	Number at risk	Survival, % (95% CI)
Robust	12	131	100.0 (100.0–100.0)
Robust	24	131	100.0 (100.0–100.0)
Robust	36	131	100.0 (100.0–100.0)
Robust	48	130	98.5 (96.4–100.0)
Robust	60	125	96.2 (92.9–99.5)
Pre-frail	12	57	100.0 (100.0–100.0)
Pre-frail	24	57	100.0 (100.0–100.0)
Pre-frail	36	55	96.5 (91.8–100.0)
Pre-frail	48	51	89.5 (81.9–97.8)
Pre-frail	60	48	84.2 (75.3–94.2)
Frail	12	116	100.0 (100.0–100.0)
Frail	24	111	95.7 (92.1–99.5)
Frail	36	108	93.1 (88.6–97.8)
Frail	48	105	90.5 (85.3–96.0)
Frail	60	103	88.8 (83.2–94.7)

Supplementary Table 3. Unadjusted RMST differences at restriction times of 24 to 60 months

Contrast	τ (months)	RMST robust	RMST comparison group	Difference (95% bootstrap CI)	P value
Pre-frail vs Robust	24	24.00	24.00	0.00 (0.00 to 0.00)	NA
Frail vs Robust	24	24.00	23.84	-0.16 (-0.31 to -0.03)	0.042
Pre-frail vs Robust	36	36.00	35.82	-0.18 (-0.47 to 0.00)	0.188
Frail vs Robust	36	36.00	35.18	-0.82 (-1.47 to -0.27)	0.008
Pre-frail vs Robust	48	47.98	47.00	-0.98 (-2.02 to -0.21)	0.036
Frail vs Robust	48	47.98	46.26	-1.73 (-2.96 to -0.70)	0.003

Contrast	τ (months)	RMST robust	RMST comparison group	Difference (95% bootstrap CI)	P value
Pre-frail vs Robust	60	59.69	57.32	-2.38 (-4.45 to -0.78)	0.012
Frail vs Robust	60	59.69	56.97	-2.73 (-4.58 to -1.09)	0.003
Non-robust vs Robust	24	24.00	23.90	-0.10 (-0.21 to -0.02)	0.039
Non-robust vs Robust	36	36.00	35.39	-0.61 (-1.06 to -0.24)	0.003
Non-robust vs Robust	48	47.98	46.50	-1.48 (-2.36 to -0.74)	<0.001
Non-robust vs Robust	60	59.69	57.08	-2.61 (-4.02 to -1.38)	<0.001

Supplementary Table 4. Full coefficients of the primary model (τ = 60 months; joint 2-df Wald test for frailty P = 0.23)

Term	Coefficient (months)	Robust SE	95% CI	P value
Intercept	68.11	6.63	55.11 to 81.12	<0.001
Pre-frail vs robust	-1.48	0.99	-3.42 to 0.45	0.134
Frail vs robust	-0.81	0.89	-2.56 to 0.93	0.361
Age, per year	-0.12	0.10	-0.33 to 0.08	0.228
Male vs female	-0.12	0.80	-1.68 to 1.45	0.883
TNM stage II vs I	-1.85	1.08	-3.97 to 0.27	0.088
TNM stage III vs I	-2.75	1.93	-6.53 to 1.04	0.155

Supplementary Table 5. Adjusted RMST differences at restriction times of 36, 48 and 60 months

τ (months)	Model	Contrast	Adjusted RMST difference (95% CI)	P value
36	Unadjusted	Pre-frail vs robust	-0.18 (-0.43 to 0.08)	0.185
36	Unadjusted	Frail vs robust	-0.82 (-1.43 to -0.21)	0.008
36	Unadjusted	Non-robust vs	-0.61 (-1.03 to	0.004

τ (months)	Model	Contrast	Adjusted RMST difference (95% CI)	P value
		robust	-0.19)	
36	Age, sex	Pre-frail vs robust	-0.01 (-0.28 to 0.27)	0.972
36	Age, sex	Frail vs robust	-0.49 (-1.02 to 0.03)	0.067
36	Age, sex	Non-robust vs robust	-0.25 (-0.53 to 0.02)	0.071
36	Age, sex, TNM stage (primary)	Pre-frail vs robust	0.07 (-0.25 to 0.39)	0.680
36	Age, sex, TNM stage (primary)	Frail vs robust	-0.31 (-0.74 to 0.12)	0.160
36	Age, sex, TNM stage (primary)	Non-robust vs robust	-0.12 (-0.36 to 0.13)	0.361
48	Unadjusted	Pre-frail vs robust	-0.98 (-1.90 to -0.07)	0.036
48	Unadjusted	Frail vs robust	-1.73 (-2.87 to -0.59)	0.003
48	Unadjusted	Non-robust vs robust	-1.48 (-2.30 to -0.66)	<0.001
48	Age, sex	Pre-frail vs robust	-0.70 (-1.68 to 0.29)	0.168
48	Age, sex	Frail vs robust	-1.14 (-2.24 to -0.03)	0.044
48	Age, sex	Non-robust vs robust	-0.92 (-1.66 to -0.18)	0.015
48	Age, sex, TNM stage (primary)	Pre-frail vs robust	-0.52 (-1.48 to 0.44)	0.290
48	Age, sex, TNM stage (primary)	Frail vs robust	-0.71 (-1.66 to 0.24)	0.143
48	Age, sex, TNM stage (primary)	Non-robust vs robust	-0.61 (-1.26 to 0.03)	0.063
60	Unadjusted	Pre-frail vs robust	-2.38 (-4.23 to -0.53)	0.012
60	Unadjusted	Frail vs robust	-2.73 (-4.49 to -0.97)	0.002

τ (months)	Model	Contrast	Adjusted RMST difference (95% CI)	P value
60	Unadjusted	Non-robust vs robust	-2.61 (-3.95 to -1.27)	<0.001
60	Age, sex	Pre-frail vs robust	-1.77 (-3.78 to 0.24)	0.085
60	Age, sex	Frail vs robust	-1.50 (-3.39 to 0.40)	0.122
60	Age, sex	Non-robust vs robust	-1.63 (-3.10 to -0.16)	0.030
60	Age, sex, TNM stage (primary)	Pre-frail vs robust	-1.48 (-3.42 to 0.45)	0.134
60	Age, sex, TNM stage (primary)	Frail vs robust	-0.81 (-2.56 to 0.93)	0.361
60	Age, sex, TNM stage (primary)	Non-robust vs robust	-1.16 (-2.50 to 0.19)	0.092

Supplementary Table 6. Proportional hazards assessment and time-varying effect analyses (non-robust vs robust)

Analysis	Result	P value
Scaled Schoenfeld residual test, non-robust (KM-transformed time)	$\chi^2 = 6.46$	0.011
Scaled Schoenfeld residual test, non-robust (log time)	$\chi^2 = 4.77$	0.029
Scaled Schoenfeld residual test, non-robust (untransformed time)	$\chi^2 = 5.78$	0.016
Cox model with log(time) interaction, non-robust × log(t), adjusted	coefficient = -11.67 (SE 6.74)	0.083
Conventional Cox HR, non-robust vs robust, unadjusted (average over follow-up; PH violated)	HR = 3.56 (1.35 to 9.39)	0.010
Conventional Cox HR, non-robust vs robust,	HR = 1.53 (0.49 to 4.79)	0.468

Analysis	Result	P value
adjusted for age, sex, TNM stage		
Firth-penalized Cox HR, non-robust vs robust, adjusted for age, sex, TNM stage	HR = 1.44 (0.50 to 4.79)	0.513
Piecewise HR 0–36 months, unadjusted (Firth-type bias reduction)	HR = 16.2 (0.95 to 276)	0.054
Piecewise HR 36–60 months, unadjusted (Firth-type bias reduction)	HR = 1.87 (0.69 to 5.11)	0.220
Ratio of period-specific HRs (0–36 vs 36–60), unadjusted	8.6 (0.426 to 174.9)	0.160
Piecewise HR 0–36 months, adjusted for age, sex, TNM stage	HR = 7.0 (0.42 to 116)	0.176
Piecewise HR 36–60 months, adjusted for age, sex, TNM stage	HR = 0.82 (0.27 to 2.53)	0.736
Ratio of period-specific HRs (0–36 vs 36–60), adjusted	8.5 (0.456 to 156.8)	0.152
Events (robust vs non-robust), 0–36 months	0 vs 10	
Events (robust vs non-robust), 36–60 months	5 vs 12	

Supplementary Table 7. E-values for unmeasured confounding (e.g., adjuvant therapy)

Quantity	Value
Unadjusted risk ratio for 5-year death, non-robust vs robust	3.33
E-value (point estimate)	6.12
Risk ratio adjusted for age, sex, TNM stage (modified Poisson)	1.49 (95% CI 0.41 to 5.41)
E-value (point estimate)	2.34

Quantity	Value
E-value (lower confidence limit)	1.00

Supplementary Figures

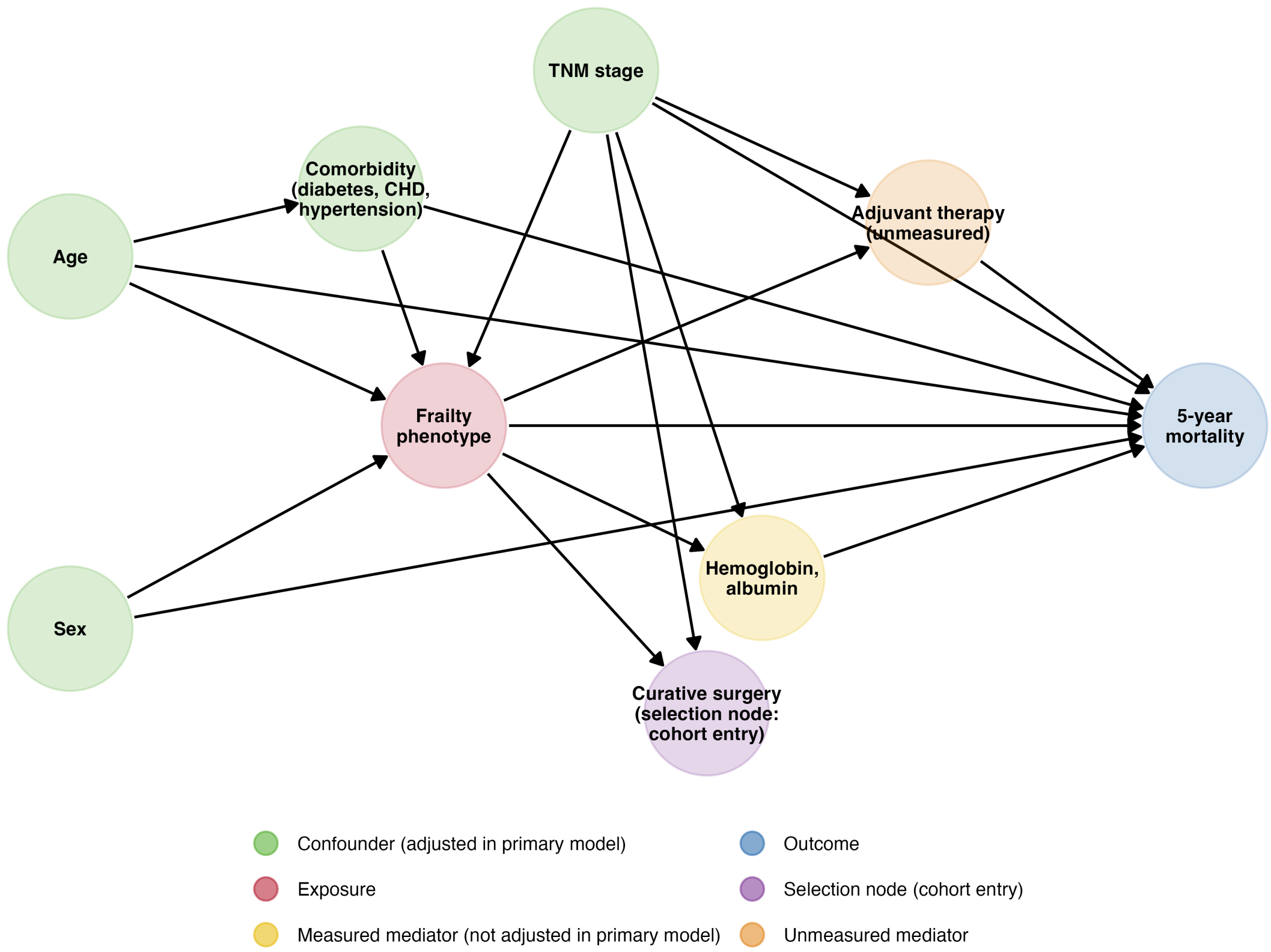

Supplementary Figure 1. Directed acyclic graph used to select covariates. Frailty phenotype is the exposure and 5-year mortality the outcome. Age, sex, comorbidity, and TNM stage are confounders and form the minimal sufficient adjustment set together with surgical selection, on which the cohort is conditioned by design. Hemoglobin and albumin are treated as measured mediators and adjuvant therapy as an unmeasured mediator.

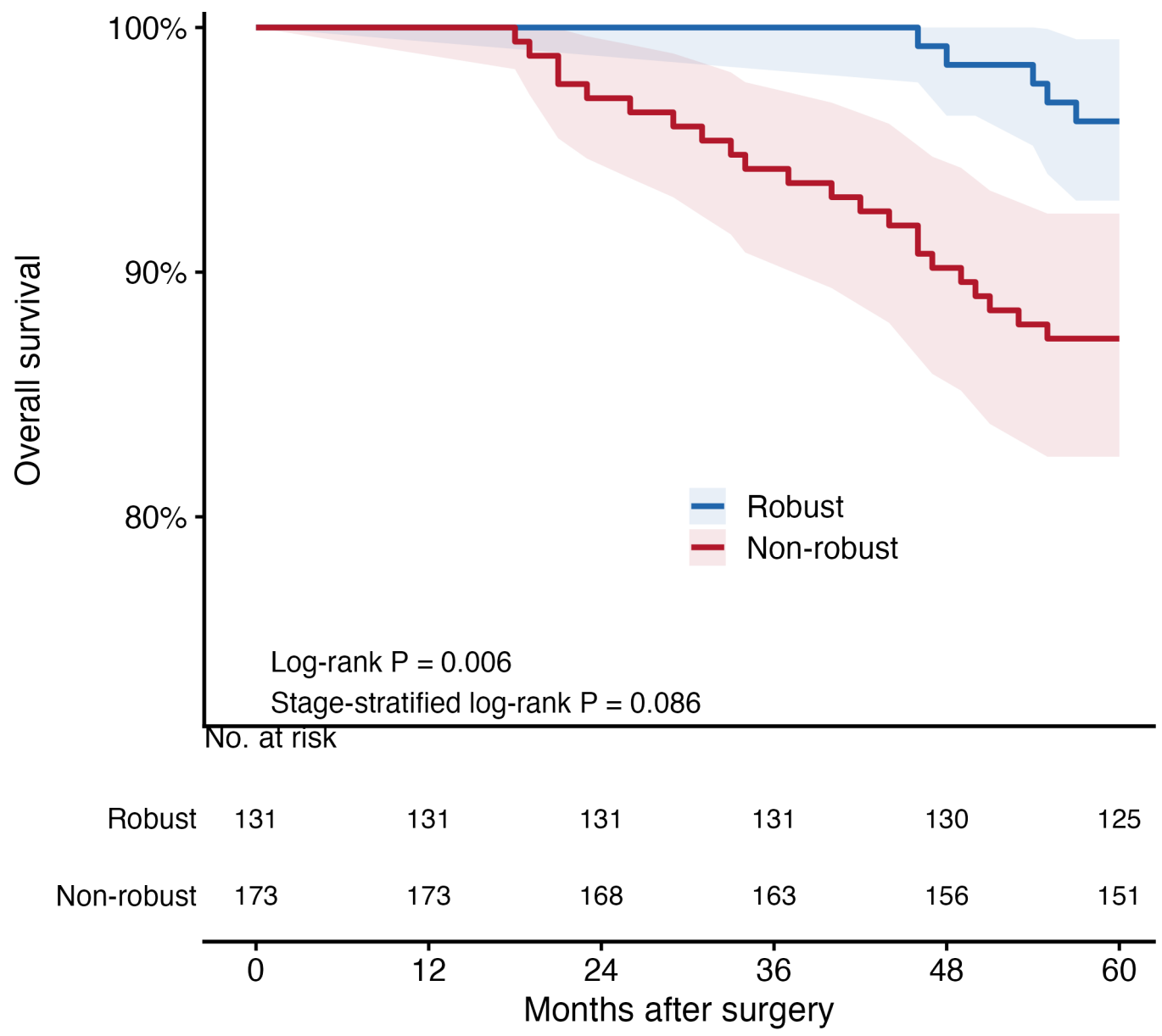

Supplementary Figure 2. Kaplan-Meier overall survival curves for robust (n=131) versus non-robust (n=173) patients. Log-rank P=0.006.

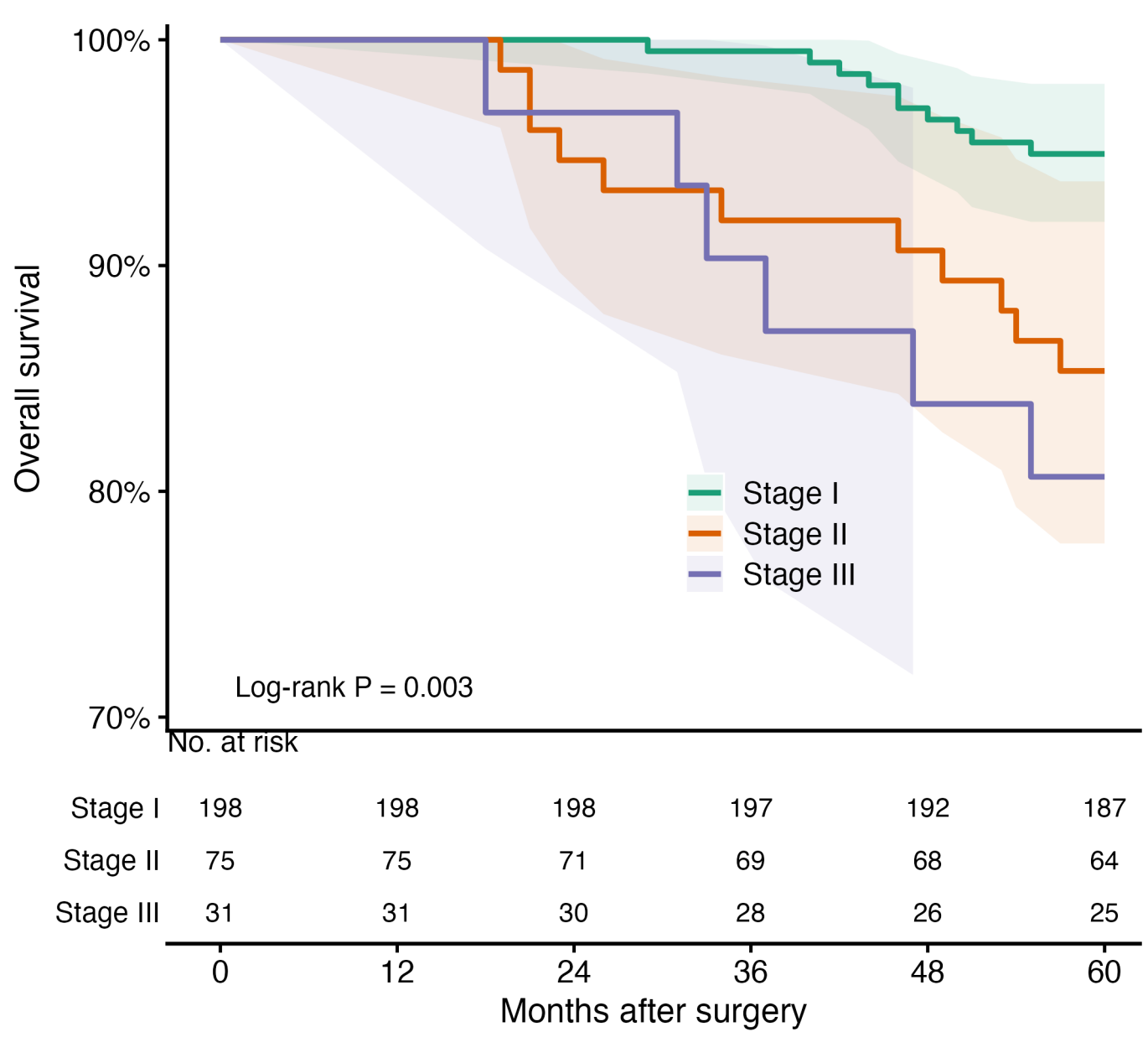

Supplementary Figure 3. Kaplan-Meier overall survival curves by TNM stage. Stage I (n=198), stage II (n=75), and stage III (n=31); log-rank P<0.001.

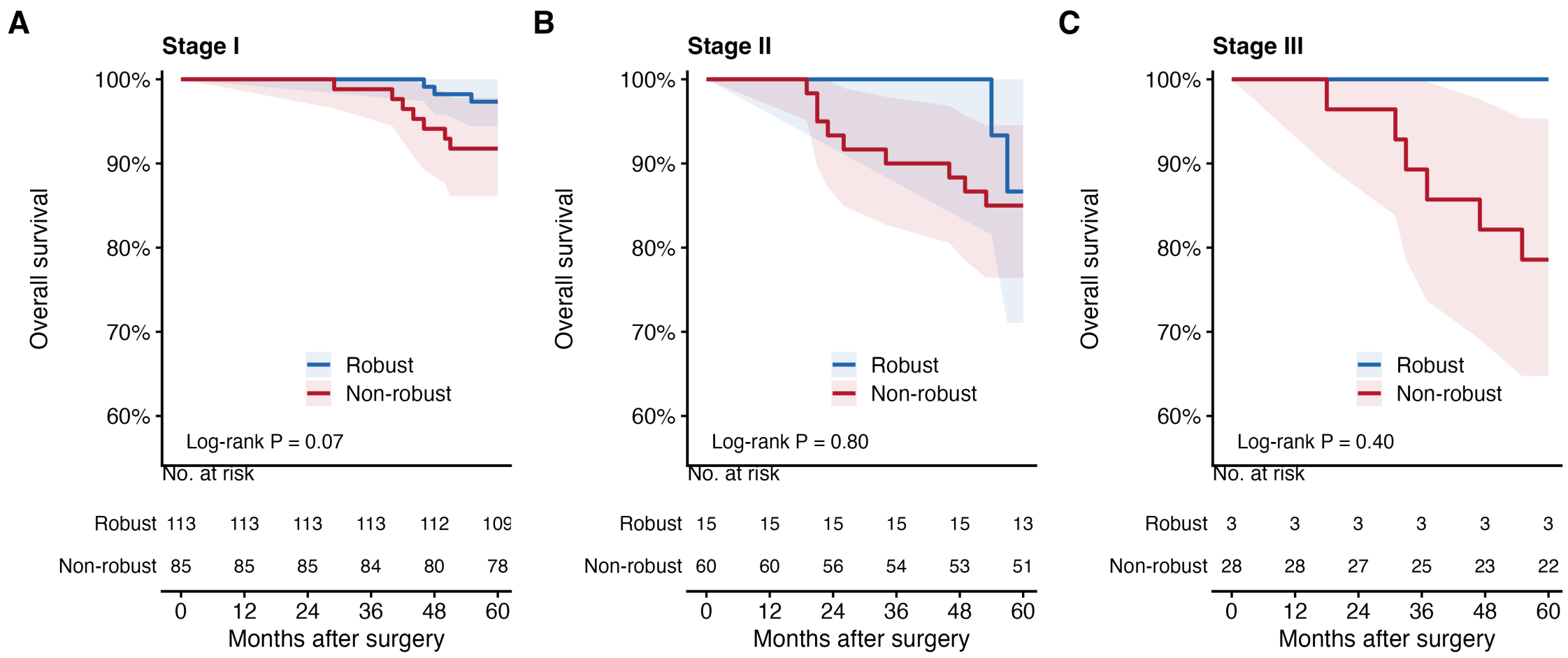

Supplementary Figure 4. Kaplan-Meier overall survival curves for robust versus non-robust patients within each TNM stage. (A) Stage I, (B) stage II, (C) stage III. Stage III contains three robust patients and the comparison in that stratum is not interpretable.

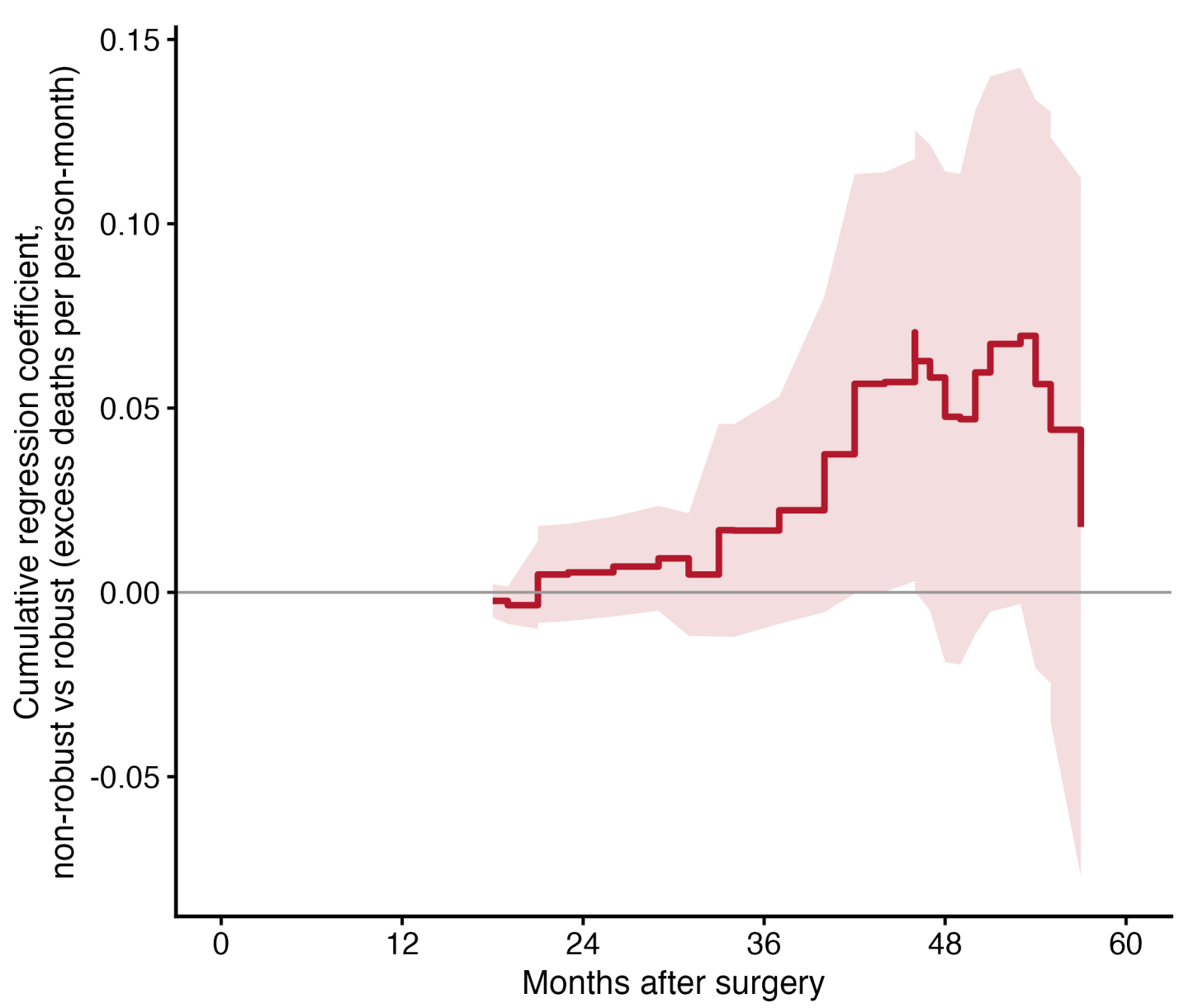

Supplementary Figure 5. Aalen additive hazards model. Cumulative regression coefficient for non-robust versus robust patients, adjusted for age, sex, and TNM stage, with 95% confidence band. The slope of the curve at any time is the excess hazard associated with non-robust status at that time.

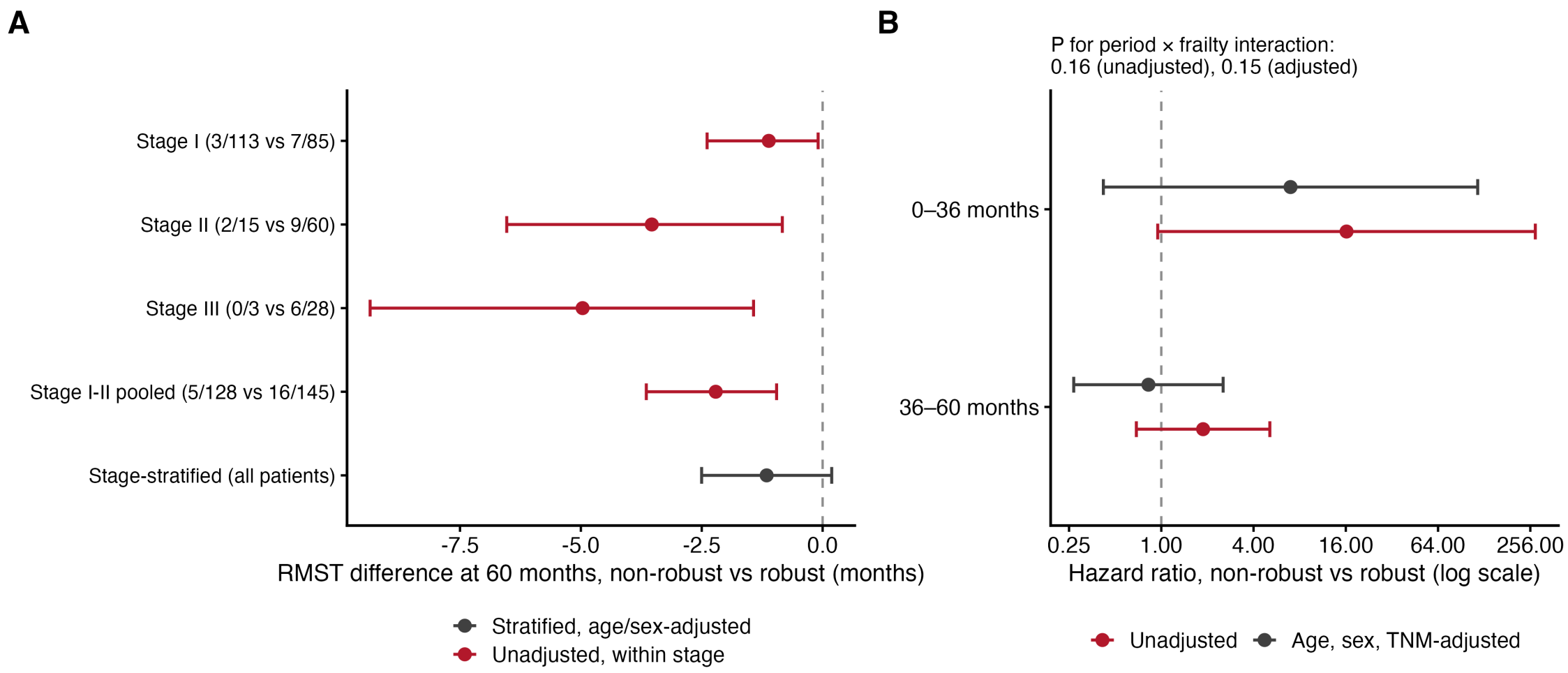

Supplementary Figure 6. Stage-specific estimates and period-specific hazard ratios. (A) Unadjusted RMST differences at 60 months for non-robust versus robust patients within each TNM stage, in stages I and II combined, and from the model using pseudo-observations computed within stage with additional adjustment for age and sex; deaths/patients are shown for the robust and non-robust groups in each stratum. (B) Hazard ratios for non-robust versus robust patients in the 0–36 and 36–60 month periods, estimated with a Firth-type bias-reduced piecewise exponential model, unadjusted and adjusted for age, sex, and TNM stage. The 0–36 month period contains no deaths among robust patients, so the estimate relies on bias reduction and is very imprecise; the period × frailty interaction is not statistically significant.
